# Supplementary material for: A new polymorphism on chromosome 6 associated with bolting tendency in sugar beet
Source: BMC Genet. 2015 Dec 7;16:142. doi: 10.1186/s12863-015-0300-2 (PMC4672520; doi:10.1186/s12863-015-0300-2)
Supplement: Additional file 2: Table S2. — Sequences of the designed primers and TaqMan probes for detection of the SNP183. (DOC 28 kb) [file 12863_2015_300_MOESM2_ESM.doc]

**Supplementary material S2.** Sequences of the designed primers and TaqMan probes for detection of the SNP183.

| Assay ID | Forward Primer Seq. | Reverse Primer Seq. | Reporter 1 Dye | Reporter 1 Sequence | Reporter 2 Dye | Reporter 2 Sequence |
| --- | --- | --- | --- | --- | --- | --- |
| SNP183 | TTGATCAATGTCCGACGTTTAGGTT | AGCTAGCATGCATGCATGACA | VIC | ATTTGAGTATCGTAAAAGAA | FAM | TGAGTATCGTGAAAGAA |
